# Sociodemographic characteristics that impact southeastern US consumers' awareness and concern about highly pathogenic avian influenza in dairy products

**DOI:** 10.3168/jdsc.2025-0849

**Published:** 2025-09-17

**Authors:** Alicia Rihn, Nama Raj Bhusal, Caitlin Zaring Weir, Elizabeth Eckelkamp

**Affiliations:** 1 Department of Agricultural and Resource Economics, University of Tennessee, Knoxville, TN 37996; 2 Department of Animal Sciences, University of Tennessee, Knoxville, TN 37996

## Abstract

The highly pathogenic avian influenza H5N1 (HPAI) has recently been identified in the dairy industry in the United States. Research has addressed consumer behavior in the context of HPAI among poultry products and birds, but fewer studies have addressed consumer awareness in the dairy context. Given recent HPAI infections in dairy herds and viral loads in fluid milk, a better understanding of consumer awareness and concern could aid in future efforts to inform the public. An online survey of 9 states in the southeastern US was conducted to elicit consumer awareness of HPAI and concern in the context of dairy products. Whether HPAI influenced purchasing behavior of dairy products was also assessed. A total of 5,290 respondents were surveyed with 14.7% (n = 775) having heard of HPAI in the dairy industry. Binary and ordered probit models were used to analyze the data. People who had young children (<12 yr old), farm backgrounds, higher incomes, or lived in metropolitan areas, suburbs, or small towns were more likely to be aware of HPAI than individuals without these attributes. Females were less likely to be aware relative to other genders. Concern over HPAI in dairy products was heightened for participants with young children, with farm backgrounds, or those who live in metropolitan areas (relative to rural areas). Among aware participants, females exhibited a decreased dairy product purchase likelihood in the context of HPAI.

## Highlights

• Only 15% of surveyed southeastern US consumers were aware of HPAI in dairy.
• People with children, farm backgrounds, or urban residences were more concerned.
• Awareness of HPAI minimally affected dairy buying habits among surveyed consumers.
• Targeted risk communication on dairy safety and animal health is highlighted.

## Graphical Abstract

**Figure:**
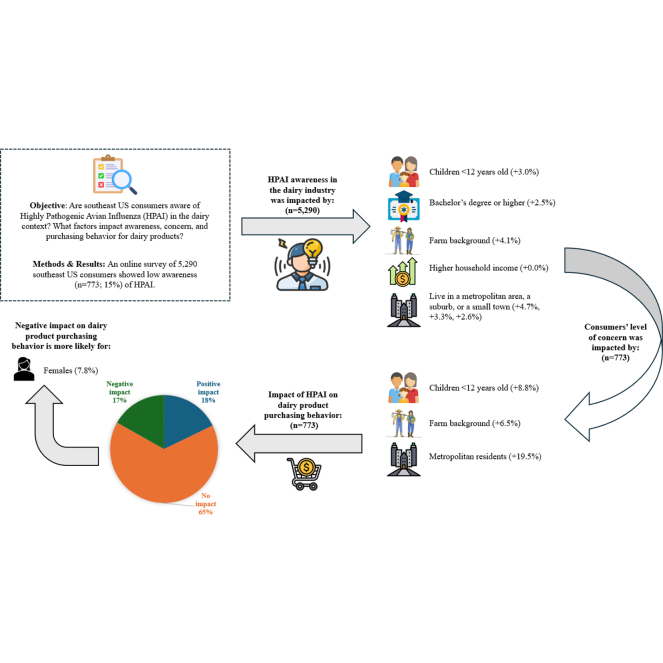
**Summary:** In a survey of 5,290 southeastern US participants, only 15% were aware of highly pathogenic avian influenza (HPAI) in the context of the dairy industry. Awareness was higher among the respondents with children under the age of 12, individuals holding at least a bachelor's degree, respondents with farm backgrounds, individuals with higher incomes, and respondents who live in metropolitan areas, suburbs, or small towns. Among the aware group, concern levels were significantly higher in households with children under 12 (+8.8%), those with a farm background (+6.5%), and metropolitan residents (+19.5%). Among aware participants, 66% reported no impact of HPAI on their dairy purchasing behavior, while 18% reported a positive impact, and 17% had a negative impact. These findings suggest that, although awareness remains low, concern varies by demographic factors and may influence dairy purchase decisions in specific consumer groups.

Highly pathogenic avian influenza (**HPAI**) is an influenza type A virus that is deadly to domestic poultry and has been detected in commercial and backyard flocks, wild birds, livestock, and domestic and wild mammals (Korteweg and Gu, 2008; USDA Animal and Plant Health Inspection Service, 2025). Highly pathogenic avian influenza research has primarily been conducted in the poultry industry and has been detected worldwide (Olsen et al., 2006; Kobayashi et al., 2017). It can negatively impact human and animal health and agriculture profitability (Gutiérrez et al., 2009; Rehman et al., 2022). Humans can become infected if they are exposed to infected animals or eat improperly prepared animal products from infected animals (Yamamoto et al., 2017; CDC, 2025a). Between 2003 and 2025, there have been 465 cases of human infection across 6 countries with a fatality rate of 67.3% (WHO, 2025). Many studies address HPAI in the context of birds (see review by Ayuti et al., 2024); however, very few address consumer behavior in industries outside of the poultry industry. This is of interest given the recent increase in dairy herds testing positive for HPAI (CDC, 2025b). Although the risk of HPAI through dairy animal exposure is low (CDC, 2025a,b), as information is shared through media outlets with end consumers, consumer purchasing behavior may be affected (Wen et al., 2019). Here, we conducted an initial study addressing the sociodemographic characteristics of southeastern US consumers that influence their awareness of HPAI in the context of the dairy industry and how that affects their level of concern and dairy product purchasing behavior.

In the United States, the first confirmed case of HPAI in dairy cattle occurred in spring of 2024 in New Mexico (USDA, 2024). Currently, in the United States, 17 states have experienced HPAI outbreaks in dairy cows, with 1,072 herds affected (CDC, 2025a). The HPAI virus in dairy cattle can affect producer profitability. In a Michigan dairy herd case study, Rodriguez et al. (2025) found a 32% incidence rate during a 45-d outbreak. During that time, milk production decreased by 5.7% (324 kg of milk/cow), SCC increased by 3-fold, and DMI consumption decreased, resulting in added costs of approximately $504 per cow. With transmission occurring across species and high potential economic consequences, researchers call for a holistic approach to handling the challenges related to HPAI in dairy cattle (Lombard et al., 2025; Owusu and Sanad, 2025). Beyond herd health, Suarez et al. (2025) tested retail dairy products (cheeses, butter, ice cream, fluid milk) and found viral RNA in 17.4% of the tested products, but no live virus. Further analysis found that the RNA closely aligned with HPAI in dairy herds and that pasteurization killed the virus, rendering the products safe for consumption.

From the consumer behavior perspective, many studies address HPAI in the poultry industry (Hsu et al., 2008; Wen et al., 2019). A survey addressing poultry consumption of Taiwanese customers determined that HPAI knowledge and perceived risk influence consumption behavior (Hsu et al., 2008). As knowledge increased, perceived risk increased, and customers engaged in behaviors to minimize exposure (i.e., avoided crowds and birds). Individuals with high perceived risk of HPAI exposure decreased their consumption of chicken. Similarly, a survey of Chinese consumers found that customers avoided certain poultry products as their uncertainty and risk perceptions of HPAI increased (Wen et al., 2019). Interestingly, they found that media reports had the greatest impact on public opinion and purchasing behavior during HPAI outbreaks. This is of interest given the increased attention by mass media to HPAI cases in the dairy industry and potential implications in the general public's purchasing and consumption behavior of dairy products (AAAS, 2025; NPR, 2025).

Although dairy products produced using pasteurized milk from infected herds are safe for consumption (Suarez et al., 2025), the increased prevalence of HPAI in dairy herds (CDC, 2025a) and media attention to this information (AAAS, 2025; NPR, 2025) means there is a need to identify how the general public is interpreting this information and how that is affecting their behavior toward dairy products. Based on this evidence, the research objective of this study was to assess southeastern US consumers' awareness of HPAI in the context of the dairy industry and determine sociodemographic factors affecting their awareness, level of concern, and purchase likelihood for dairy products. Results are applicable to dairy industry stakeholders as they consider strategies to communicate with end customers about HPAI in the dairy industry.

An online survey instrument was used to address the research objective. The survey was conducted in Qualtrics LLC (Provo, UT) and an online panel was purchased from the same company. Data collection occurred between September and December 2024. The survey instrument consisted of an informed consent form, screening questions, state identity questions, a discrete choice experiment, dairy purchasing behavior questions, HPAI perception questions, and sociodemographic questions. Participants were screened to ensure they were adults (≥18 yr old), that they or someone in their household consumed dairy products, that they had food shopping responsibilities in their household, and that they resided in one of the 9 states of interest (i.e., Alabama, Arkansas, Florida, Georgia, Louisiana, Mississippi, South Carolina, Virginia, West Virginia). Individuals who passed the screening questions proceeded to the remainder of the survey. All study procedures and protocols were approved and deemed exempt by the Institutional Review Board (IRB-23–07515-XM).

For this analysis, the HPAI perception and sociodemographic questions were used. From a list of 8 foodborne illnesses, participants selected those that they had heard of in relation to dairy products. Answer options included brucellosis, campylobacteriosis, cryptosporidiosis, *Escherichia coli*, enterohemorrhagic *Escherichia coli* O157 (EHEC O157), highly pathogenic avian influenza (HPAI), *Listeria*, and *Salmonella*. These foodborne illnesses have been previously documented in the dairy industry (CDC, 2025a,c), meaning it is reasonable that some participants may have heard of them in the context of dairy products that could affect their perceptions of dairy product safety and purchasing behavior. The list of illnesses was provided to reduce bias where participants indicate awareness of the option of interest simply due to HPAI being the only option. If participants selected one of the foodborne illnesses, they were asked how concerned they were about that foodborne illness when considering dairy products using a 5-point Likert scale (1 = not at all concerned; 5 = extremely concerned). For participants who had heard of HPAI, they were asked how HPAI affected their purchasing behavior for dairy products with answers ranging from 1 equaling “very negative impact” to 7 equaling “very positive impact.” Participants who had not heard of HPAI did not answer the concern or purchase likelihood questions but proceeded to the remainder of the survey.

Given the distribution of answers, the concern and purchase likelihood ratings were each consolidated into 3 levels for analysis. Concern was recoded to 1 if participants selected a rating of 1 or 2 (no or slight concern) on the original 5-point scale, 2 if they selected a 3 (somewhat concerned), and 3 if they selected a 4 or 5 (high/very concerned). The purchase likelihood ratings were recoded to −1 indicating a negative impact on purchase likelihood (if participants selected 1, 2, or 3 on the original 7-point scale), 0 equaled no impact (selecting 4), and +1 equaled a positive impact (if participants selected a 5, 6, or 7).

Probit models and marginal effect estimates were used to analyze the results where awareness (model 1), concern (model 2) and purchase likelihood (model 3) were used as dependent variables. Given that the awareness dependent variable was binary (1 = aware; 0 = not aware), a probit model was estimated. Following Long and Freese (2006), the structural model can be written as

[1] $$y_{i}^{*}=x_{i}\beta +\varepsilon_{i},$$

where y* is the latent variable ranging from −∞ to ∞, *i* is the individual, ***β*** indicates the estimated coefficients of the independent variables (i.e., state of residence, sociodemographics), ***x*** is the regressor vector for individual *i*, and *ε* is the random error term. The binary dependent variable model can be expanded into *J* ordinal categories where

[2] $$y_{i}=m\;\mathrm{if}\;c_{m-1}\leq y_{i}^{*}<c_{m}\;\mathrm{for}\;m=1\;\mathrm{to}J,$$

where the thresholds of *c*_1_ through c*_J_*_−1_ are estimated and we assume *c*_0_ = −∞ and *c_J_* = ∞. For the concern and purchase likelihood models, the dependent variable is ordinal in nature, meaning an ordinal probit model is appropriate. The probability of observing *y* = *m* for given values of y* is between *c_m_*_−1_ and *c_m_* and can be expressed as

[3] *P*(*y* = *m*|***x***) = *F*(*c_m_* − ***xβ***) − *F*(*c_m_*_−1_ − ***xβ***),

where *F* is the cumulative distribution function for *ε* with Var(*ε*) = 1.

The concern and purchase likelihood ordinal probit models were estimated with the original ratings (5-level for concern; 7-level for purchase likelihood) and the new 3-level ratings. The Akaike information criterion (**AIC**) and Bayesian information criterion (**BIC**) were used to determine which exhibited a better model fit by exhibiting lower values (Mohammedet al., 2015). The AIC and BIC values indicate that the 3-level dependent variable models fit the data better than the original models. Thus, the 3-level ratings were used in the analysis. Based on the model estimates, marginal effects were generated. Only significant marginal effects are presented, with the complete model and marginal effect estimates available from the corresponding author upon request.

A total of 5,290 people completed the survey. On average 69% were female; 27% had a bachelor's degree or higher; 37% were married; 21% had a farm background; and 12% lived in metropolitan areas, 35% in the suburbs, 19% in small towns, and 30% in rural areas (Table 1). Their mean 2022 household income was $54,319. State of residence was uniform at 11% of the sample, except for West Virginia which was 10% of the sample. At the time of the study, only 15% of the people sampled were aware of HPAI in the dairy industry.

**Table 1:** Sociodemographic summary statistics of southeastern US consumers from an online survey conducted in 2024

Demographic	Definition	Total (n = 5,290)		HPAI aware (n = 775)		HPAI not aware (n = 4,515)		*P*-value^1^
		Mean	SD	Mean	SD	Mean	SD	
Female	1 = yes; 0 = otherwise	0.690	0.463	0.638	0.481	0.699	0.459	0.001***
Bachelor's+	1 = bachelor's degree or higher; 0 = otherwise	0.271	0.445	0.322	0.467	0.263	0.440	0.001***
Married	1 = yes; 0 = otherwise	0.367	0.482	0.377	0.485	0.365	0.481	0.517
Child	1 = child <12 yr old at home; 0 = otherwise	0.226	0.418	0.260	0.439	0.220	0.414	0.015**
Farm background	1 = yes; 0 = otherwise	0.214	0.410	0.252	0.435	0.208	0.406	0.005***
Income	2022 household income in $1,000s	54.319	45.766	60.525	50.201	53.263	44.889	0.000***
Metropolitan	1 = lives in metropolitan area; 0 = otherwise	0.119	0.324	0.140	0.348	0.115	0.320	0.048*
Suburban	1 = lives in suburban area; 0 = otherwise	0.348	0.476	0.375	0.484	0.343	0.475	0.091
Small town	1 = lives in small town; 0 = otherwise	0.191	0.393	0.185	0.389	0.192	0.394	0.657
Rural	1 = lives in rural area; 0 = otherwise	0.299	0.458	0.246	0.431	0.308	0.462	0.001***

1 *P*-values were calculated using pairwise *t*-tests to identify significant differences between HPAI aware and not aware groups. Asterisks indicate significance at the 1% (***), 5% (**), and 10% (*) levels.

Key demographic differences between the aware and not aware groups were estimated. Significant differences were noted in gender, education level, presence of children, farm background, household income, metropolitan and rural residence, and state of residence (Mississippi, Virginia). The aware group consisted of a smaller portion of females, people who live in rural areas, and Mississippi residents while having higher education levels, more children, farm backgrounds, higher incomes, metropolitan residency, and residency in Virginia.

Data were analyzed to summarize participants' awareness of foodborne illnesses in the context of the dairy industry. Most participants had heard of *E. coli* (73%), S*almonella* (70.6%), and *Listeria* (51.5%). Few participants had heard of HPAI (15%), brucellosis (10%), EHEC O157 (8%), campylobacteriosis (5%), and cryptosporidiosis (4.6%) in the dairy industry. On average, participants had heard of 2.4 (SD = 1.31) of the listed foodborne illnesses. A Spearman correlation was conducted between participants' awareness of the different foodborne illnesses. Positive correlations existed between participants' awareness of the different foodborne illnesses, demonstrating that if they were aware of one, they were likely aware of the other illnesses. For example, there were positive significant correlations between being aware of HPAI and being aware of brucellosis, campylobacteriosis, cryptosporidiosis, EHEC O157, *Listeria*, and S*almonella*. For the more well-known foodborne illnesses (*E. coli* and *Salmonella*) some differences were observed. There were positive correlations between participants being aware of *E. coli* in the dairy industry and being aware of *Listeria*, *Salmonella*, *Campylobacteriosis*, and *Cryptosporidiosis*. For salmonella, positive correlations existed between awareness of *Salmonella* in the dairy industry and *E. coli*, HPAI, and *Listeria*.

Southeastern US consumers' level of concern about different foodborne illnesses in dairy products is presented in Table 2. Participants exhibited the highest level of concern for EHEC O157 (mean = 2.02; SD = 0.875), followed by *Salmonella*, HPAI, *Listeria*, *E. coli*, campylobacteriosis, cryptosporidiosis, and brucellosis. Ratings were not significantly different across the foodborne illnesses.

**Table 2:** Southeastern US consumers' level of concern about different foodborne illnesses (n = 5,290)

Illness	No. of observations	3-Point scale^1^	
		Mean	SD
Brucellosis	531	1.789	0.858
Campylobacteriosis	268	1.873	0.873
Cryptosporidiosis	243	1.856	0.881
*Escherichia coli*	3,881	1.919	0.892
Enterohemorrhagic *Escherichia coli*O157 (EHEC O157)	426	2.018	0.875
Highly pathogenic avian influenza	775	1.981	0.886
*Listeria*	2,718	1.919	0.897
*Salmonella*	3,727	2.005	0.899

1 The 3 scale points correspond to 1 = not at all or slightly concerned, 2 = somewhat concerned, and 3 = very or extremely concerned.

Table 3 presents the significant marginal effect estimates based on the probit models. Only significant results are presented and the full model estimates are available from the corresponding author upon request. Several demographic characteristics had an effect on participants' awareness (model 1). If the participant had a bachelor's degree or higher, they had a 2.5% increased probability of being aware of HPAI in dairy products. Having children in the household and a farm background increased the probability of being aware by 3.0% and 4.1%, respectively. The probability of being aware of HPAI increased by 4.7% for metropolitan residents, 3.3% for suburban residents, and 2.6% for small town residents relative to rural residents. Household income had a slight positive effect on participants' awareness. Conversely, females had a 2% decrease in probability of being aware of HPAI in dairy products relative to other genders.

**Table 3:** Probit and ordered probit model estimates^1^ and marginal effects of factors influencing southeastern US consumers' awareness, concern, and dairy purchasing decisions related to HPAI (n = 773)

Variable	Model 1: HPAI awareness		Model 2: Concern						Model 3: Impact on purchase likelihood					
	MGE (1)	SE	MGE (1)	SE	MGE (2)	SE	MGE (3)	SE	MGE (−1)	SE	MGE (0)	SE	MGE (+1)	SE
South Carolina	−0.027	0.021	0.138	0.069**	−0.003	0.003	−0.135	0.068**						
Female	−0.020	0.011*							0.078	0.024***			−0.075	0.022***
Bachelor's+	0.025	0.012**							−0.048	0.024**			0.046	0.023**
Child	0.030	0.012**	0.237	0.102**	−0.089	0.038**	0.002	0.002						
Farm background	0.041	0.012***	0.174	0.099*	−0.066	0.037*	0.001	0.002	−0.043	0.025*			0.041	0.024*
Household income	0.000	0.000**												
Metropolitan	0.047	0.017***	0.528	0.158***	−0.199	0.058***	0.004	0.005						
Suburban	0.033	0.013**												
Small town	0.026	0.015*												

1 Only significant marginal effects (MGE) and SE are presented. Full model results and MGE estimates are available from the corresponding author upon request. Model 1 was a probit model where the dependent variable equaled 1 if participants were aware of HPAI in the dairy industry. Model 2 was an ordered probit model where the dependent variable levels were 1 = no or slight concern, 2 = somewhat concerned, and 3 = high or extreme concern for HPAI in the dairy industry. Model 3 was an ordered probit model where the dependent variable levels were −1 = negative impact, 0 = no impact, and +1 = positive impact on purchase likelihood for dairy products in the context of HPAI awareness. Asterisks indicate significance at the 1% (***), 5% (**), and 10% (*) levels relative to the base variables (i.e., Florida, not female, less than a bachelor's degree, not married, no child <12 yr old, no farm background, rural).

Several demographic characteristics affected participants' level of concern regarding HPAI in dairy products (model 2; Table 3). Participants with children had an 8.8% increased probability of being in the high-concern group. If participants had a farm background, they had a 6.5% increased probability of being in the high-concern group. Metropolitan residents had a 19.5% increased probability of being in the high-concern group relative to rural residents. Conversely, individuals from South Carolina were 13.5% less likely to be in the high-concern group relative to Florida residents.

The distribution of how aware customers perceive HPAI affecting their dairy purchases was assessed. Approximately 17% of the sample indicated that HPAI would negatively affect their future dairy item purchases, while the vast majority (66%) indicated no change, and 18% indicated a positive effect. Females were 7.8% more likely to have HPAI negatively affect their dairy purchases than other genders. Conversely, individuals with a bachelor's degree or higher and those with a farm background were 4.6% and 4.1% more likely to see HPAI positively affecting their purchasing behavior for dairy products.

The presence of HPAI in the dairy industry is relatively recent (USDA, 2024) but widely publicized (AAAS, 2025; NPR, 2025), meaning there may be effects on consumer behavior as awareness increases (Wen et al., 2019). Understanding who is aware of HPAI and how they respond provides key insights as the dairy industry considers future communication strategies with end customers.

Southeastern US consumers exhibited low awareness for HPAI in the dairy industry context. This is counter to research that demonstrates high consumer awareness (90%) but low knowledge (25%) when considering HPAI in general (Ipsos, 2025). Furthermore, when considering awareness of other foodborne illnesses, participants exhibited higher ratings for more historically common foodborne illnesses (*E. coli*, *Salmonella*, *Listeria*; CDC, 2025a,c). The prevalence of customer awareness of *E. coli*, *Salmonella*, and *Listeria* may be linked to recent outbreaks in raw dairy products (US FDA, 2024; CDC, 2025d) or due to outbreaks of these illnesses from other food products (e.g., melons; Canning et al., 2025) causing increased awareness in general. Supporting evidence from this study demonstrated that awareness of one foodborne illness improved the probability of being aware of other foodborne illnesses. This may indicate that as information is available, foodborne illnesses become more of a focal point for customers and may influence their consumption and purchasing behavior, similar to Wen et al. (2019).

Demographics affected participants' level of awareness and concern related to HPAI in the dairy industry. Females and participants from rural areas exhibited lower awareness. Education, young children, and farm backgrounds increased awareness. Interestingly, not all demographics that influenced awareness translated to heightened concern. Only the presence of children, a farm background, and living in a metropolitan area increased participants' level of concern of HPAI in dairy products. The presence of children may be due to parents considering the safety and health of their children. Consequently, they may attend to health information more than individuals without children. Regarding farm backgrounds, given that people often are infected with HPAI due to direct exposure to infected animals (CDC, 2025a; WHO, 2025), it is possible that individuals working or having worked on a farm may be more aware and concerned about HPAI due to animal exposure.

When considering purchasing behavior for dairy products, most participants (66%) indicated that HPAI in the dairy industry has no effect on their purchasing behavior. This is counter to research conducted in the poultry industry, which demonstrates decreased consumption behavior (Hsu et al., 2008; Wen et al., 2019). This discrepancy may be related to heightened awareness within the poultry industry and the presence of the disease for a longer duration (Ayuti et al., 2024). Alternatively, it may be due to pasteurization of dairy products killing foodborne illnesses (including HPAI), which renders the products safe for consumption (Suarez et al., 2025), whereas in the poultry industry, improperly prepared food can cause infection (Yamamoto et al., 2017). Interestingly, individuals with higher levels of education and those with a farm background indicated increased dairy purchasing behavior if they were aware of HPAI in the dairy industry. This may imply that educated consumers or those with farm backgrounds are aware of the safety precautions, pasteurization process or both, of dairy products.

Generally, the results demonstrated low awareness of HPAI and low impacts on purchasing behavior in the dairy industry among southeastern US consumers. As information becomes more available, the industry may benefit from communicating the food safety processes and the safety of dairy products regardless of whether the milk is from an infected herd or not. Messaging related to animal health and welfare may bolster this information while generating value for customers (Wolf and Tonsor, 2017).

Although the results provide insights into consumer behavior related to HPAI in the dairy industry, several limitations need to be acknowledged. First, the study was conducted on a targeted sample of southeastern US dairy product purchasers. As such, the results may not be applicable in other regions or individuals. Future studies could expand the sampling procedures to account for both limits. Second, the data represented self-reported hypothetical metrics. Comparing the results with scanner data at times of intense media blasts related to HPAI in the dairy industry could aid in better understanding the effect of mass media on purchasing behavior in this context. Last, although participants were directed to only consider HPAI in the dairy context, other factors may have come into consideration. Future work could delve into additional factors (e.g., psychological drivers, information treatments) to assess the influence of HPAI on consumer behavior related to dairy products.
